# The Experimental and Modeling Study on the Thermodynamic Equilibrium Hydrate Formation Pressure of Helium-Rich Natural Gas in the Presence of Tetrahydrofuran

**DOI:** 10.3390/molecules29204827

**Published:** 2024-10-11

**Authors:** Zengqi Liu, Guangqi Zhang, Fangfang Lu, Qiyuan Ren, Zhen Xu, Shiguang Fan, Qiang Sun, Yiwei Wang, Xuqiang Guo

**Affiliations:** 1 State Key Laboratory of Heavy Oil Processing, China University of Petroleum-Beijing at Karamay, Karamay 834000, China; liuzq@cupk.edu.cn (Z.L.); guoxq@cup.edu.cn (X.G.); 2 State Key Laboratory of Heavy Oil Processing, China University of Petroleum (Beijing), Beijing 102249, Chinasunq@cup.edu.cn (Q.S.)

**Keywords:** helium, methane, gas hydrate, phase equilibrium, model

## Abstract

Hydrate-based gas separation (HBGS) has good potential in the separation of helium from helium-rich natural gas. HBGS should be carried out under a pressure higher than the thermodynamic equilibrium hydrate formation pressure (*P_eq_*) to ensure the formation of hydrate so that the accurate prediction of *P_eq_* is the basis of the determination of HBGS pressure. In this work, the *P_eq_* of the helium-rich natural gases with different helium contents (1 mol%, 10 mol%, and 50 mol%) in gas and different tetrahydrofuran (THF) contents (5 wt%, 10 wt%, and 19 wt%) in liquid at different temperatures were experimentally investigated through the isothermal pressure search method. A new thermodynamic model was proposed to predict the *P_eq_* of helium-rich natural gas. This model can quantitatively describe the effects of THF and helium on *P_eq_*, and it predicts the *P_eq_* of the helium-rich natural gases in this work accurately. The average relative deviation of the model is less than 3%. This model can guide the determination of the operating condition of the HBGS of helium-rich natural gas.

## 1. Introduction

Helium (He) is widely used in aerospace, cryogenic science, electronic production, medicine treatment, instrument manufacturing, and other high-tech industries [1] due to its stable chemical properties [2], low boiling temperature (4.2 K), and small radius (0.26 nm) [3]. The global annual demand of helium exceeded 3000 tons in 2020 [4], and it continuously increases. The helium recovery from natural gas (NG) is the only method for industrial-scale helium production [5]. The mole fraction of helium in NG is extremely low. NG is treated as helium-rich natural gas (HNG) and holds industrial value when the mole fraction of the helium in NG is higher than 0.3 mol% [6].

Though many technologies were proposed to purify the helium from HNG, pressure swing adsorption (PSA), membrane, and cryogenic separation are the main separation methods for the industrial production of helium. PSA and membrane are used to produce helium of high purity with small-scale industrial production [7]. Pressure swing adsorption (PSA) requires lower energy consumption, but the recovery of helium limits the use of PSA for low-purity helium separation [8]. The membranes have high selectivity and lower energy consumption, but a larger surface area is required for membranes leading to high capital cost for low-purity helium separation [9]. Trace amounts of hydrogen sulfide (H_2_S) NGs are harmful to SPA and membrane [10]. Pressure swing adsorption (PSA) [8] and membrane separation [9] are used as supplementary steps to purify high-purity helium gas from crude helium gas (the mixture of CH_4_ and helium), which is produced by cryogenic separation because those two methods cannot efficiently separate helium from the gas with low helium content. Cryogenic separation has high helium recovery but causes high energy consumption for purifying high-purity helium gas. About 90% of helium is recovered by cryogenic separation [11]. Therefore, a separation method with low energy consumption and low equipment costs is expected.

Hydrate-based gas separation (HBGS) is an accepted separation method with low energy consumption, low equipment costs, and is not negatively affected by H_2_S [10]. HBGS is suitable for separating the small gases when the diameter of the gas molecules is much smaller than the diameter of the water molecule cage in hydrate, like hydrogen. HBGS has been used to separate hydrogen from fluid catalytic cracking dry gas (H_2_ + CH_4_ + C_2_H_6_ + CO_2_) [10], hydrogenation tail gas (H_2_ + CH_4_ + C_2_H6 + C_3_H_8_) [12,13], and hydrogen-compressed natural gas (H_2_+ CH_4_ + C_2_H_6_ + C_3_H_8_) [14]. Han et al. [14] recovered hydrogen with a purity of 98.73% from a feed gas comprising H_2_ (30.0 mol%), CH_4_ (63.0 mol%), C_2_H_6_ (4.9 mol%), and C_3_H_8_ (2.1 mol%) by using three-stage-HBGS in the presence of tetrahydrofuran (THF). Helium, which has a smaller molecular diameter than hydrogen, is the most suitable target gas for HBGS. Therefore, helium recovery from HNGs by using HBGS is an accepted separation method.

**Figure 1:**
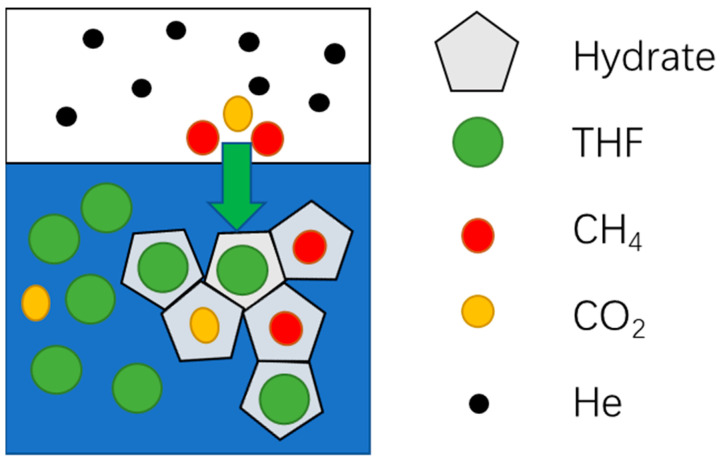
The schematic of differences in equilibrium hydrate formation. The bule background is the liquid phase, and the white background is the gas phase.

Gas hydrates are non-stoichiometric crystals formed by water and guest molecules. The guest molecules are gas and liquid molecules which are trapped in cages of water molecules, like methane (CH_4_) [15], carbon dioxide (CO_2_) [16], and THF [17]. Different gases require different thermodynamic conditions for hydrate formation [10]. The gas compositions of HNGs from different sources are different. Generally, the HNG consists of helium, CH_4_, CO_2_, H_2_S, and other hydrocarbons [18,19]. During the HBGS process of the HNG, the gases that have smaller molecular diameters (such as helium and hydrogen) can hardly form hydrate and are enriched in the gas phase, while the other gases form hydrate and are enriched in the hydrate phase [20], which is shown in Figure 1.

The application of HBGS is limited by the requirement of hydrate formation conditions with low temperatures and high pressures [21]. Since high pressure leads to high energy consumption, how to decrease the thermodynamic equilibrium hydrate formation pressure (*P_eq_*) is crucial to the HBGS of the helium from HNGs in the industry. *P_eq_* can be shifted toward lower pressures by using thermodynamic promoters [22], such as THF. THF is a thermodynamic promoter which is soluble in water and can significantly reduce *P_eq_* [23]. The *P_eq_* of hydrate formation in the presence of THF is much lower than that of hydrate formation in the absence of THF. The effect of THF in the HBGS of hydrogen is better than other promoters [10,12,13,14]. Helium is similar to hydrogen in hydrate formation; for instance, it has a similar high *P_eq_* [24]. Therefore, THF was chosen as a thermodynamic promoter in this work.

Since HBGS separates gas mixtures by forming hydrate, the HBGS pressure should be higher than *P_eq_* to ensure the formation of hydrate [24]. It is crucial to accurately predict the *P_eq_* of different HNGs with different THF solutions. However, no model can ensure the accurate prediction of the *P_eq_* of the corresponding systems, and the experimental data that can support the establishment of the prediction model is very little. The van der Waals and Platteeuw model [25,26,27] and Chen–Guo model [28] are the two most-used models for predicting the *P_eq_* of THF hydrate with NGs. In the Chen–Guo model, hydrate formation is a two-step process: the quasi-chemical reaction equilibrium of basic cages and the physical adsorption equilibrium of gas molecules in the link cages, which is different from the van der Waals and Platteeuw model. For sII hydrate (24 Gas · 136 H_2_O), 8 larger cages (5^12^6^4^, a hexahedron with 12 pentagons and 4 hexagons) are the large cages (or basic cage) and 16 smaller cages (5^12^, a dodecahedron with 12 pentagons) are the small cages (linked cages) [28]. The basic cages are filled with calculated quasi-chemical reaction equilibrium; this is different from the van der Waals and Platteeuw model, which is calculated using Langmuir adsorption. The Chen–Guo model has significant improvements in the prediction accuracy of hydrate formation conditions [29,30]. Both the Chen–Guo model and van der Waals–Platteeuw model rely on fitting the Langmuir adsorption, so the experimental study on *P_eq_* is the prerequisite for modeling.

In this work, the *P_eq_* of HNGS in the presence of THF was experimentally investigated and was accurately predicted to verify the feasibility of the HBGS process of helium from HNGs. The effects of the THF content in liquid and helium content in gas on the *P_eq_* were quantitatively described by the model in this work. To reveal the effect of helium on hydrate formation, the binary gas (CH_4_ and helium) and ternary gas (CH_4_, helium, and CO_2_) were used in this work. This is the first work that accounted for the effects of helium in a gas mixture on *P_eq_* in a thermodynamic model. The model can quantitatively describe the effects of THF and helium on *P_eq_*, and it predicts the *P_eq_* of the helium-rich natural gases in this work accurately. The new thermodynamic model and experimental data in this work can help to further understand the effects of helium and THF on *P_eq_* and can guide the prediction and control of the HBGS of helium from HNGs.

## 2. Model

In this work, we proposed a thermodynamic model based on the Chen–Guo model for *P_eq_* [20,29]. The model used the Patel–Teja (PT) equation of state (EoS) [31] to calculate the fugacity of CH_4_, CO_2_, and helium, which are provided in Appendix A. Calculation for the Fugacity of Gases, and used the Wilson activity coefficient model to calculate the activity of water and the fugacity of THF [28]. The Wilson activity coefficient model is a simple and accurate method for gas–liquid phase equilibria of nonionic solutions [32], which is suitable for the CH_4_-THF-water system [28]. This is the first thermodynamic model that accounted for the effects of helium in a gas mixture on *P_eq_*. The model can quantitatively describe the effects of THF and helium on *P_eq_*, and it predicts the *P_eq_* of the model’s helium-rich natural gases in this work accurately.

**Figure 2:**
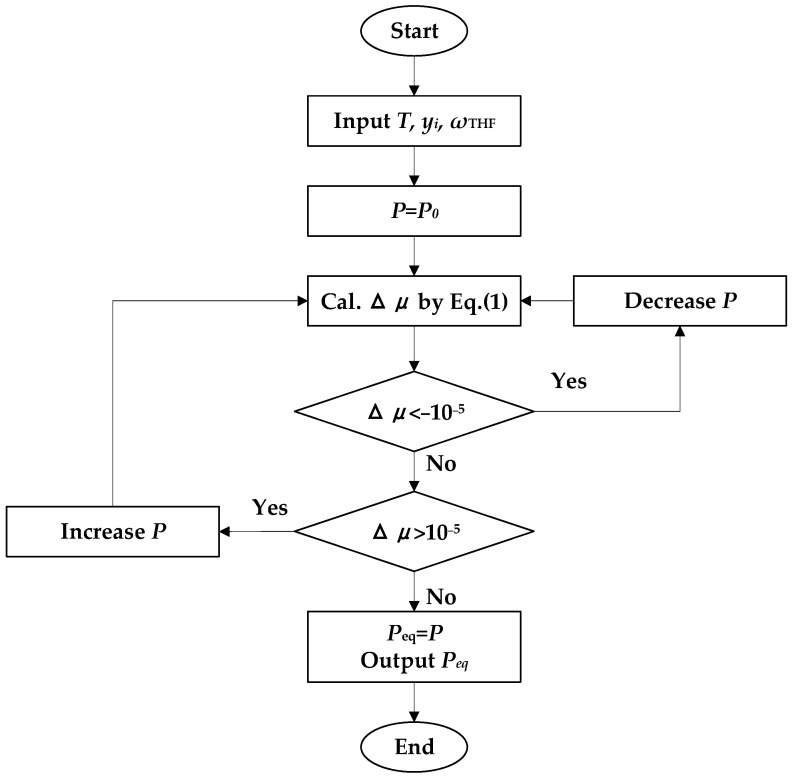
Schematic diagram of the prediction of *P_eq._* $y_{i}$ is the mole fractions of gases in the gas phase and *w* is the mass fraction of THF.

The phase equilibrium conditions of the systems in this work are determined by the difference in chemical potential between phases (Δμ). When the Δμ = 0, the pressure of the system is the *P_eq_* [15]. The procedure for predicting *P_eq_* is illustrated in Figure 2.

The Δ*µ* can be calculated as follows [28,29]:

(1) $$\Delta \mu =\mathrm{RT}\left[\lambda_{2}\;\ln\frac{f_{THF}^{H}}{f_{THF}}+\sum_{i}\lambda_{1}\ln\left(1-\theta_{i}\right)\right]$$

where $\theta_{i}$ is the occupation fraction of the linked cages in hydrates filled by gas and *i* is the CH_4_, CO_2_, and helium, respectively. $f_{THF}$ and $f_{THF}^{H}$ are the fugacity of THF in the liquid phase and the basic hydrate under the experimental condition, respectively. R is the gas constant (8.314 J·K^−1^·mol^−1^). $\lambda_{1}$ is the ratio of the linked-cage number to the water-molecule number. $\lambda_{2}$ is the ratio of the basic-cage number to the water-molecule number. $\lambda_{1}$ and $\lambda_{2}$ are determined by the hydrate structure (sII). For THF hydrate (sII), $\lambda_{1}$ is 2/17 and $\lambda_{2}$ is 1/17 [20,29].

Based on the Langmuir adsorption theory, the occupation fraction of the linked cages in hydrates $\theta_{i}$ can be expressed as follows [29]:

(2) $$\theta_{i}=\frac{f_{i}C_{i}}{1+\sum_{i}\left(f_{i}C_{i}\right)}$$

where $f_{i}$ is the fugacity of CH_4_, CO_2_, and helium calculated by PT EOS. $C_{i}$ is the Langmuir constant of CH_4_, CO_2_, and helium correlated as an Antoine-type equation [29]:

(3) $$C_{i}=X^{\prime }\exp\left(\frac{Y^{\prime }}{T-Z^{\prime }}\right)$$

where $X^{\prime }$, $Y^{\prime }$, and $Z^{\prime }$ are the Antoine parameters. The parameters for CH_4_, CO_2_, and helium are fitted by the experimental data of this work, as shown in Table 1.

Based on the Chen–Guo model, the fugacity of tetrahydrofuran in the hydrate phases ($f_{THF}^{H}$) is calculated as follows [33]:

(4) $$f_{THF}^{H}=f_{T}^{H}\left(T\right)\cdot \exp\left(\frac{\beta P}{T}\right){\cdot \alpha }_{w}^{-\frac{1}{\lambda_{2}}}$$

where *β* is the parameter of hydrate structure, which is 10.244 K/MPa [20,29]. $\alpha_{w}$ is the activity of the water calculated by the Wilson model. $f_{T,j}^{H}\left(T\right)$ is a faction of temperature in the Chen–Guo model, which can be written as follows [33]:

(5) $$f_{T}^{H}(T)=exp(\frac{\sum_{i}A_{i}\theta_{i}}{T})\cdot A^{\prime }exp(\frac{B^{\prime }}{T-C^{\prime }})$$

where $A^{\prime }$, $B^{\prime }$, and $C^{\prime }$ are the Antoine parameters. For THF hydrate, $A^{\prime }$ is 1.80 × 10^24^ Pa, $B^{\prime }$ is −2.0 × 10^4^ K, and $C^{\prime }$ is −130 K. The $A_{i}$ is the binary interaction parameter between gas *i* and THF in the hydrate. For THF hydrate, $A_{{CH}_{4}}$ is 300, $A_{{CO}_{2}}$ is 300, and $A_{He}$ is 100. All the parameters are fitted by *P_eq_* from the literature and this study.

The activity of water in solution was calculated by the Wilson model to be written as follows [28]:

(6) $$ln\gamma_{1}=-ln\left(x_{1}+\Lambda_{12}x_{2}\right)+x_{2}\left(\frac{\Lambda_{12}}{x_{1}+\Lambda_{12}x_{2}}-\frac{\Lambda_{21}}{x_{2}+\Lambda_{21}x_{1}}\right)$$

(7) $$ln\gamma_{2}=-ln\left(x_{2}+\Lambda_{21}x_{1}\right)+x_{1}\left(\frac{\Lambda_{21}}{x_{2}+\Lambda_{21}x_{1}}-\frac{\Lambda_{12}}{x_{1}+\Lambda_{12}x_{2}}\right)$$

(8) $$\Lambda_{12}=\frac{v_{2}^{L}}{v_{1}^{L}}exp\left(-\frac{\lambda_{12}-\lambda_{11}}{RT}\right)$$

(9) $$\Lambda_{21}=\frac{v_{1}^{L}}{v_{2}^{L}}exp\left(-\frac{\lambda_{21}-\lambda_{22}}{RT}\right)$$

(10) $$\alpha_{w}=\gamma_{1}x_{1}$$

where $x_{1}$ and $x_{2}$ are the mole fractions of water and THF, respectively. $v_{2}^{L}$ and $v_{1}^{L}$ are the mole volumes of water and THF, respectively. The mole volumes of water and THF can be calculated by fitting polynomials from the literature [32]. $\gamma_{1}$ and $\gamma_{2}$ are the activity coefficients of water and THF, respectively. $\lambda_{12}-\lambda_{11}$ is 1865.2097 J/mol and $\lambda_{21}-\lambda_{22}$ is 1927.6307 J/mol according to the literature [34].

The fugacity of THF in liquid is corrected by polynomials as follows:

(11) $$f_{THF}=\gamma_{2}x_{2}P_{2}^{sat}exp(\frac{v_{2}^{L}(p-P_{2}^{sat})}{RT})f_{cor}$$

(12) $$f_{cor}=-8.7774w^{2}+0.444w+1.3176$$

where $P_{2}^{sat}$ is the saturated vapor pressure of THF. w is the mass fraction of THF in the solution. $f_{cor}$ is fitted by *P_eq_* from the literature and this study. The effects of $f_{cor}$ are discussed in Section 4.

Average relative deviation (ARD), goodness of fit (GF), and standard deviation (SD) are used to calculate the deviation between experimental (exp) and predicted (pre) *P_eq_*.

(13) $$ARD=\sum_{\mathrm{ii}\;}^{n}\;\left|\frac{P_{\mathrm{eq},\;\exp,\mathrm{ii}\;}-P_{\mathrm{eq},\;\mathrm{pre},\mathrm{ii}\;}}{P_{\mathrm{eq},\;\exp,\mathrm{ii}\;}}\right|/n\cdot 100\%$$

(14) $$GF=1-\frac{\sum_{ii}^{n}\;{\left(P_{eq,exp,ii}-P_{eq,pre,ii}\right)}^{2}}{\sum_{ii}^{n}\;{\left(P_{eq,\;\exp,\mathrm{ii}\;}-\sum_{ii}^{nn}\;P_{eq,pre,ii}/n\right)}^{2}}$$

(15) $$SD=\sqrt{\frac{\sum_{ii\;}^{n}\;{\left(\frac{P_{eq,\;exp,ii\;}-P_{eq,\;pre,ii\;}}{P_{eq,\;exp,ii\;}}\right)}^{2}}{n}}$$

## 3. Results

To show the effects of THF on hydrate formation, the initial concentrations of THF in aqueous solutions are 5.0 wt%, 10.0 wt%, and 19.0 wt%. In 19 wt% THF aqueous solution, the mole ratio of THF to water is 1/17, which is the same as the mole ratio of THF to the water of sII hydrate ($\lambda_{2}$ = 1/17).

To show the effects of helium on hydrate formation, the mixture of CH_4_ and helium was used in this work. The mole ratios of CH_4_-helium in the binary gas mixture are 99.0/1.0 (HNG1), 90.0/10.0 (HNG2), and 50.0/50.0 (HNG3). The actual HNG from Aksu, Xinjiang (Aksu gas) consists primarily of CH_4_, CO_2_, helium, and other hydrocarbons. The total mole fraction of other hydrocarbons in Aksu gas is less than 1 mol%. The Aksu gas is simplified to a ternary gas system to confirm the HBGS is not negatively affected by the acidic gas components (CO_2_). The composition of the simplified natural gas (HNG4) is CH_4_ 95.6 mol%, CO_2_ 4.0 mol%, and helium 0.4 mol%.

### 3.1. Single Gas System

**Figure 3:**
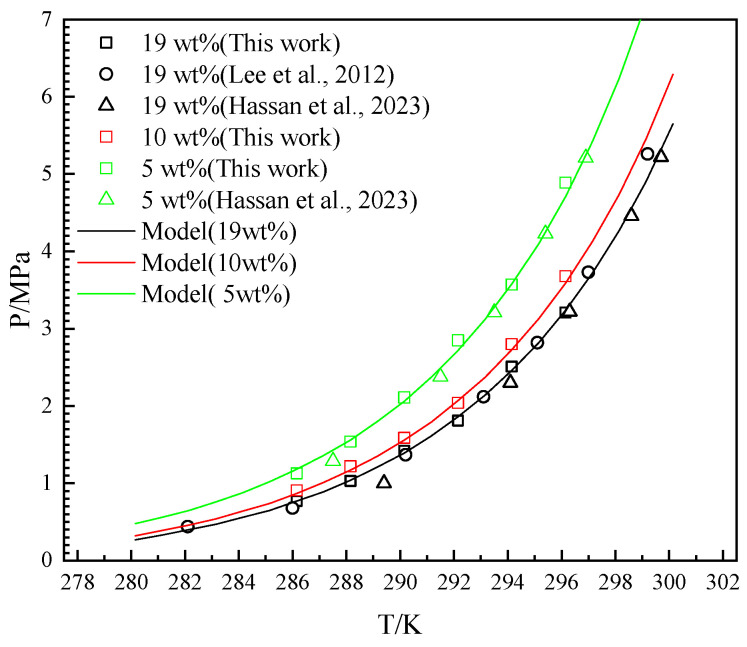
Equilibrium hydrate formation conditions with experimental, literature, and predicted data for CH_4_-THF-water system. (Lee et al., 2012) stands for [21] and (Hassan et al., 2023) stands for [35].

The accuracy of the experimental methods and devices used in this work was quantitatively investigated through a comparison with literature data [21,35] and the Chen–Guo model of THF-CH_4_ hydrate [34]. The experimental results were measured over a temperature range of 286.15–296.15 K in the presence of 5 wt%, 10 wt%, and 19 wt% THF in the aqueous solution. The experimental and the predicted results are depicted in Figure 3.

As illustrated in Figure 3, the *P_eq_* increased with the increase in temperature from 282.1 K to 299.7 K in every gas–THF system. The *P_eq_* decreased with the increase in the initial concentration of THF in aqueous solutions. The difference between the *P_eq_* of 10 wt% and the *P_eq_* of 19 wt% is less than the difference between the *P_eq_* of 10 wt% and the *P_eq_* of 5 wt%. The difference between the *P_eq_* of different THF solutions increases in temperature. The model in this work describes the trend well, and the experimental results in this work are consistent with the literature data [21,35]. The ARD between the Chen–Guo model and the literature data [21,35] is 5.2%, while the ARD between the Chen–Guo model and the experimental result of this work is 2.6%. The GF between the Chen–Guo model and the literature data [21,35] is 0.997, while the GF between the Chen–Guo model and the experimental result of this work is 0.998. The SD between the Chen–Guo model and the literature data [21,35] is 0.123, while the SD between the Chen–Guo model and the experimental result of this work is 0.070. The ARD and SD of the literature data [21,35] are higher than the experimental results of this work because the literature data are taken from different publications with different experimental apparatuses and different methods. It can be found that some data deviate (like 289.4 K and 1.00 MPa at 19 wt% THF and 299.2 K and 5.26 MPa at 19 wt% THF) significantly from other data, which is the other reason for the larger ARD and SD. It confirms the accuracy of the experimental apparatus and methods used in this work.

### 3.2. Binary Gas System

**Figure 4:**
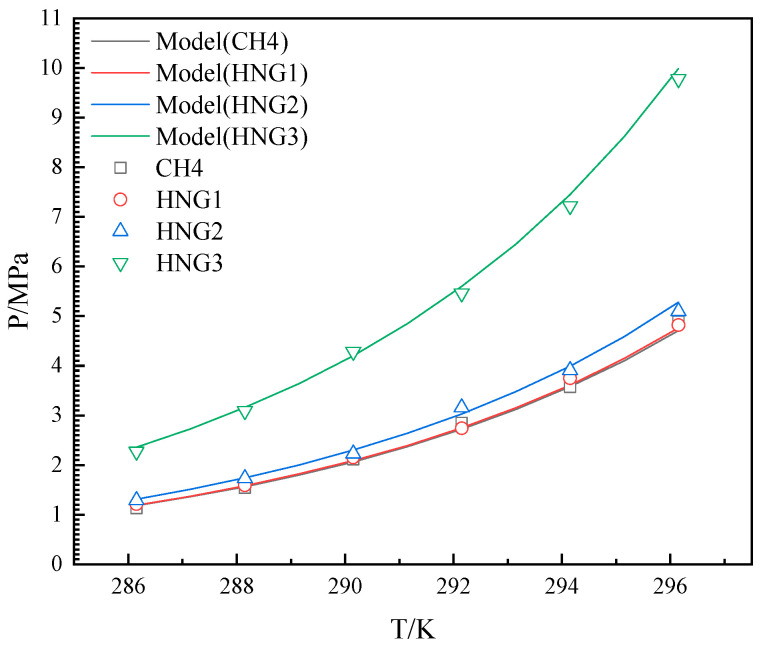
Equilibrium hydrate formation conditions with experimental and predicted data for CH4 and HNG1-HNG3 in the presence of 5 wt% THF.

**Figure 5:**
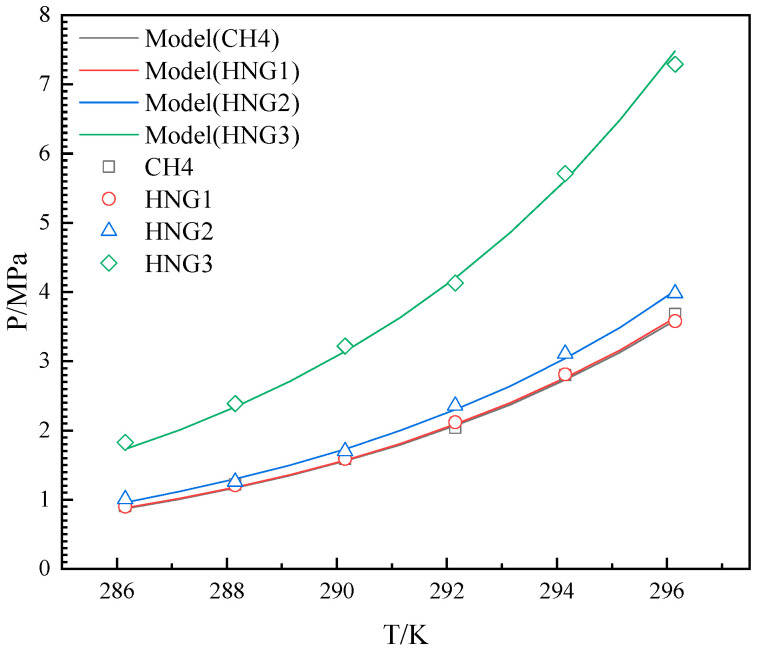
Equilibrium hydrate formation conditions with experimental, literature, and predicted data for CH4 and HNG1-HNG3 in the presence of 10 wt% THF.

**Figure 6:**
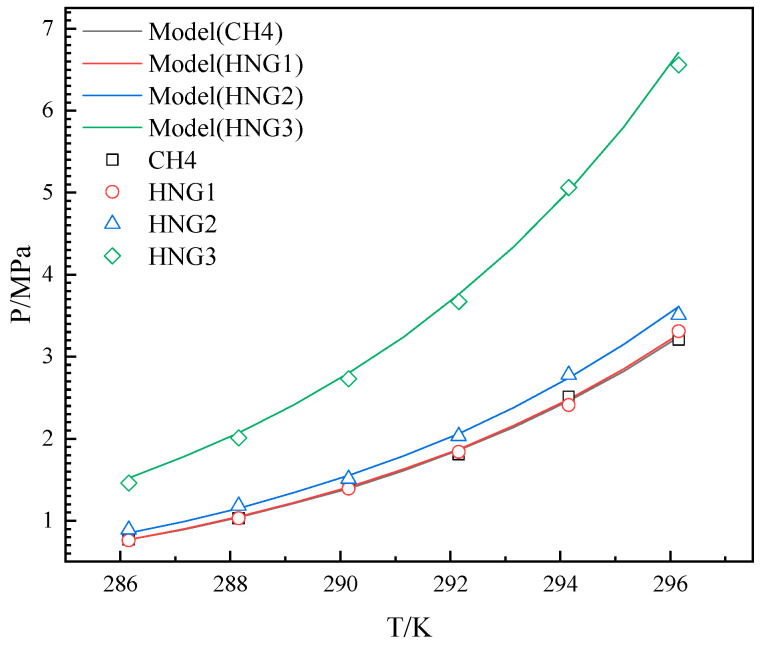
Equilibrium hydrate formation conditions with experimental data, literature, and predicted data for CH4 and HNG1-HNG3 in the presence of 19 wt% THF.

The experimental results were measured over a temperature range of 286.15 K–296.15 K for the HNGs-THF-water system. The experimental and predicted results for HNGs are depicted in Figure 4, Figure 5 and Figure 6. The experimental and predicted results for pure CH_4_ are also shown in Figure 4, Figure 5 and Figure 6, as contrasted with gas mixtures.

As illustrated in Figure 4, Figure 5 and Figure 6, the *P_eq_* increased with the increase in temperature from 286.15 K to 296.15 K in the presence of different THF concentrations. The *P_eq_* increased with the increase in the mole fraction of helium in the gas mixture (HNG1, HNG2, and HNG3) in each initial concentration of THF in aqueous solutions. The difference between the *P_eq_* of pure CH_4_ and HNG1 (99.0 mol% CH_4_) is less than 0.1 MPa in each initial concentration of THF in aqueous solutions. The HNG1 and pure CH_4_ have no significant difference in the *P_eq_* of pure CH_4_. The *P_eq_* is decreased with the increase in the initial concentration of THF in aqueous solutions with different mole fractions of helium in the gas mixture (HNG1, HNG2, and HNG3). The difference in *P_eq_* between 10 wt% and 19 wt% is less than the difference in *P_eq_* between 10 wt% and 5 wt%. The promoting effect of THF on *P_eq_* is nonlinear with the initial concentration of THF in aqueous THF solutions. The two effects of helium and THF are discussed in Section 4. The ARDs and GF between experimental and predicted *P_eq_* are listed in Table 2. All the ARDs for HNGs-THF-water systems are less than 3%, and the GFs for HNGs-THF-water systems are more than 0.998. The maximum value of SD is 0.151 for the HNG3-5 wt% THF system. This confirms that the accuracy of the model in this work meets the application of HBGS.

### 3.3. Ternary Gas System

**Figure 7:**
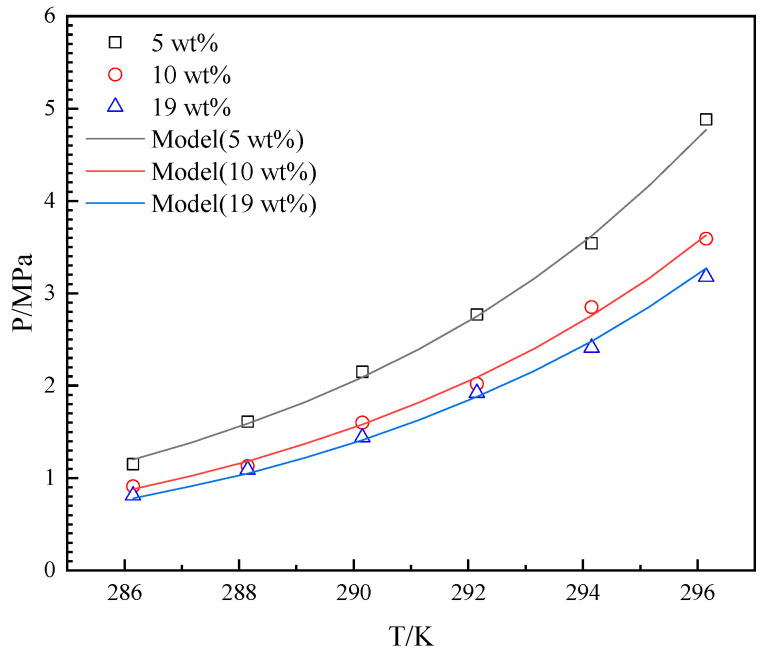
Equilibrium hydrate formation conditions with experimental data and predicted data for HNG4 in the presence of 5, 10, and 19 wt% THF.

The experimental results were measured over a temperature range of 286.15 K–296.15 K for the HNG4-THF-water system. The experimental and predicted results for HNG4 are depicted in Figure 7.

As illustrated in Figure 7, the *P_eq_* increased with the increase in temperature from 286.15 K to 296.15 K in the presence of different THF concentrations. The *P_eq_* increased with the increase in the initial concentration of THF in the aqueous solution. The promoting effect of THF on *P_eq_* is nonlinear with the initial concentration of THF in aqueous solutions of THF, which has the same tendency with CH_4_-helium-THF-water systems. As listed in Table 2. All the ARDs for HNG4 systems are less than 3%, GFs for HNGs-THF-water systems are more than 0.998, and SDs for HNG4 systems are less than 0.065. This confirms that the accuracy of the model in this work meets the prediction of HBGS.

## 4. Discussion

### 4.1. The Effects of Helium

**Figure 8:**
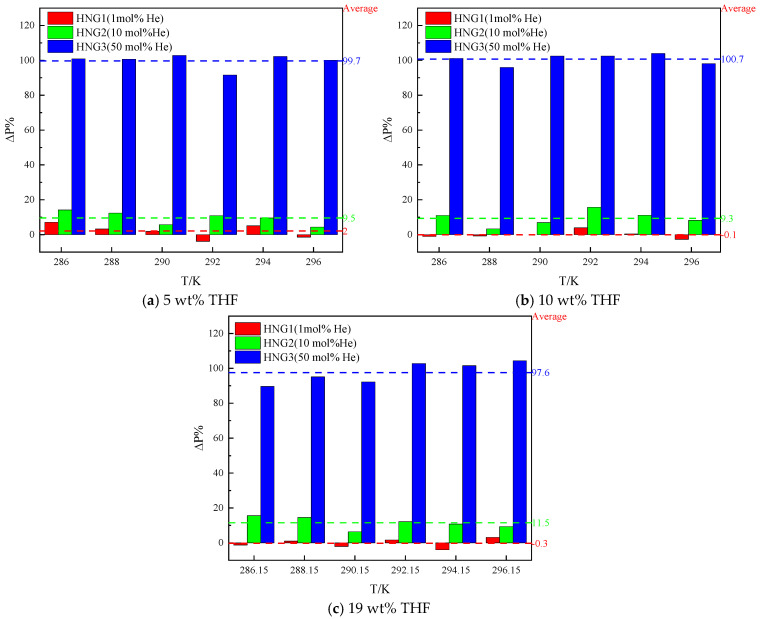
The differences in *P_eq_* between pure CH_4_ and HNG1–HNG3 in the presence of THF.

To quantify the effect of helium on *P_eq_*, the difference in *P_eq_* (∆*P*%) between the pure CH_4_ and HNGs (HNG1~HNG3) were proposed as follows:

(16) $$∆P\%=\frac{P_{HNG}-P_{{CH}_{4}}}{P_{{CH}_{4}}}\times 100\%$$

where $P_{HNG}$ and $P_{{CH}_{4}}$ are the *P_eq_* for HNG and pure CH_4_ at the same temperature and in presence of the same initial concentration of THF. The results of ∆P% in the 19 wt% THF for HNG1, HNG2, and HNG3 are shown in Figure 8.

As illustrated in Figure 8, the increase in *P_eq_* is positively correlated with the increase in mole fractions of helium in the HNGs. The difference in *P_eq_* between CH_4_ and HNG1 is not significant. The average of ∆*P*% for HNG1 is 2.0% for 5 wt% THF, −0.1% for 10 wt% THF, and −0.3% for 19 wt% THF. However, the mole fractions of helium in HNGs increase to 10 mol% (HNG2) from 1 mol% (HNG1), and the difference in *P_eq_* between CH_4_ and HNG1 increases to around 10%. The average of ∆*P*% for HNG2 is 9.5% (for 5 wt% THF), 9.3% for 10 wt% THF, and −11.5% for 19 wt% THF. When the mole fraction of helium in HNGs increases to 50 mol% (HNG2), the difference in *P_eq_* between CH_4_ and HNG1 increases to around 100% of *P_eq_* for CH_4_. The average of ∆*P*% for HNG2 is 99.7% for 5 wt% THF, 100.7% for 10 wt% THF, and 97.6% for 19 wt% THF. It can be inferred that helium almost does not participate in the formation of hydrate. As the mole fraction of helium in the gas phase increases from HNG1 to HNG3, helium dilutes the mole fraction of CH_4_ in the gas phase and reduces the fugacity of CH_4_, thus increasing the *P_eq_* of hydrate formation.

### 4.2. The Effects of THF

**Figure 9:**
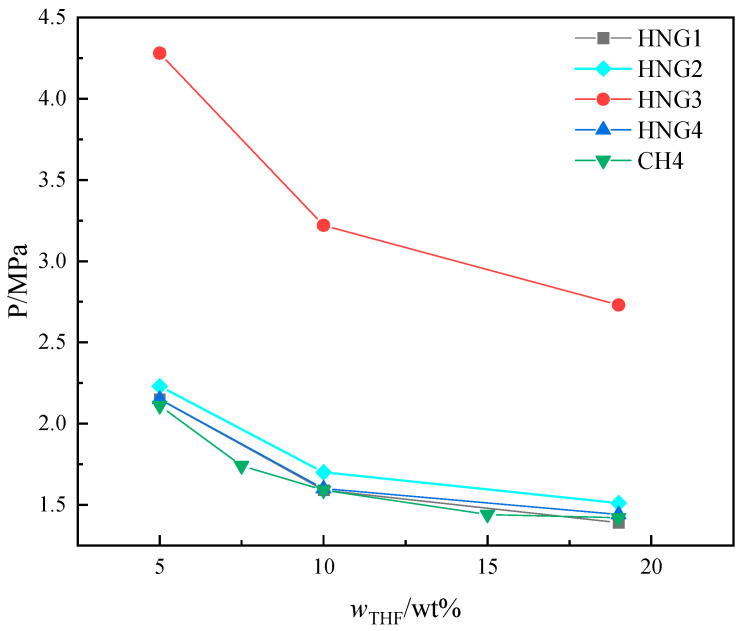
The experimental results for CH_4_ and HNGs in the presence of different concentrations of THF.

The experimental results for CH_4_ and HNGs with different initial THF concentration systems in 290.15 K are shown in Figure 9. To better obtain the tendency of the *P_eq_* with the different initial THF concentrations, the experimental results of *P_eq_* for CH_4_ with more initial concentrations of THF (7.5 wt% and 15 wt%) were supplied.

As illustrated in Figure 9, the promoting effect of THF on *P_eq_* is nonlinear with the concentration of THF in aqueous solutions. When the initial concentration of THF in aqueous solution is less than 15 wt%, the promoting effect of THF on *P_eq_* increases with an increase in the initial concentration of THF in aqueous solution. The 15 wt% and 19 wt% THF solutions have no significant difference in *P_eq_* of pure CH_4_. The data of *P_eq_* of binary gas systems (CH_4_ + helium) was used to correct the fugacity of THF in Equation (12) to match the effects on *P_eq_.* The introduction of Equation (12) represents no significant difference in the promotion effects of THF on *P_eq_* at high concentrations (15 wt%~19 wt%).

### 4.3. The Effects on P_eq_ in the Model

According to the effects of helium and THF on *P_eq_*, the Chen–Guo model has been modified to adapt to the effects. Helium almost does not participate in the formation of hydrate. As the mole fraction of helium in the gas phase increases, helium dilutes the mole fraction of CH_4_ in the gas phase and reduces the fugacity of CH_4_, thus increasing the *P_eq_* of hydrate formation. Therefore, the Langmuir constant of helium ($C_{3}$) in Equation (3) should be much smaller than that of CH_4_ ($C_{1}$) at the same temperature. In the model of this work, the $C_{3}$ is the 1.5% of $C_{1}$ at 290.15 K calculated by Equation (3). The fugacity of THF is corrected by quadratic polynomial to express how the promoting effect varies with THF concentration in Equation (12). The expression can help further understand the effects of THF on *P_eq_* and can guide the control of THF concentrations in other THF-containing HBGS.

## 5. Materials and Methods

The experimental gases, HNGs, are the gas mixture of CH_4_–helium and CH_4_–CO_2_–helium. The mole ratio of CH_4_–helium (±0.05 mol%) in the binary gas mixture is 99.0/1.0 (HNG1), 90.0/10.0 (HNG2), and 50.0/50.0 (HNG3). The mole ratio of CH_4_–CO_2_–helium (±0.05 mol%) in the ternary gas mixture is 95.6/4.0/0.4 (HNG4). The HNGs were provided by Beijing Yongsheng Gas Industry Company (Beijing, China). THF (purity ≥ 99%) was provided by Shanghai Denou Chemical Company (Shanghai, China). The deionized water (18 × 10^6^ Ω·cm) and THF were weighed by an electronic balance (±0.1 mg).

**Figure 10:**
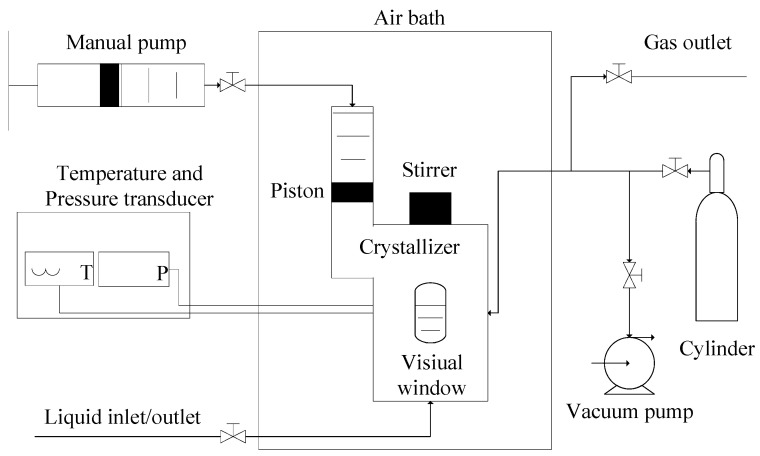
Schematic diagram of experimental apparatus for the measurements of the equilibrium hydrate formation conditions.

Figure 10 shows the experimental apparatus in this work, which is the same as that in our previous works [15,16]. The temperature range of the crystallizer is from 253.15 to 323.15 K, which is adjusted by air bath. The maximum pressure of the crystallizer is 20.00 MPa. The volume of the crystallizer was adjusted by using a manual pump with a maximum volume of 465.0 mL. The uncertainties of the measured pressure and measured temperature are ±0.005 MPa and ±0.05 K, respectively.

**Figure 11:**
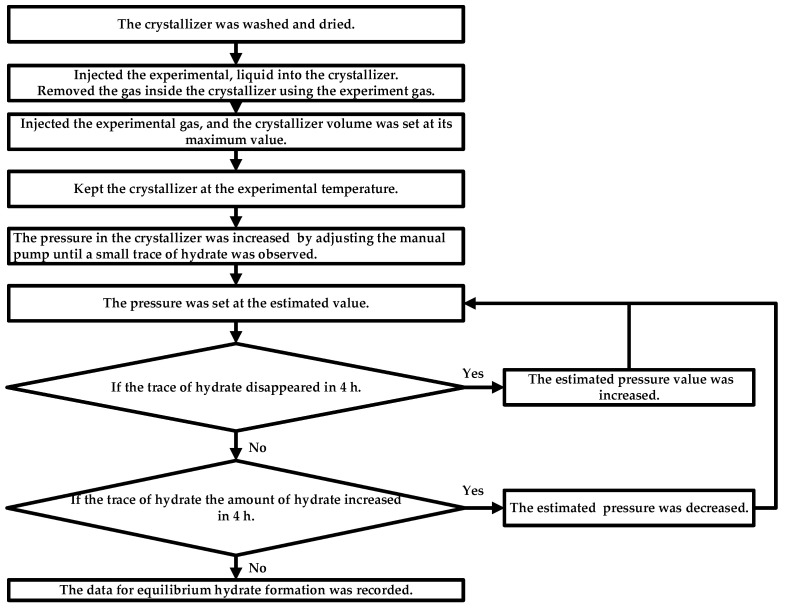
The experimental procedure of investigating the *P_eq_* through the isothermal pressure search method.

The pressure search method was used to measure the *P_eq_* [36]. The values of *P_eq_* were measured for different HNGs-liquid systems. The initial concentration of THF was set at 0, 10 wt%, and 19 wt%. The experimental procedure is the same as that in our previous works [15,16] shown in Figure 11. The amount of each feed in the experiment is controlled by volume. The volume of experimental liquid is 40.0 mL and the uncertainty in volume is ±0.05 mL. The volume of the experimental crystallizer is 465.0 mL (40.0 mL of experimental liquid and 425.0 mL of experimental gas in the crystallizer), the uncertainty in volume is ±0.05 mL. All the mole ratios of the gases (HNG1-4) are the same as in the cylinder. The uncertainty of the mole ratio is ±0.05 mol%, which is equal to the volume ratio ±0.05 vol% under standard condition (273.15 K and 101.325 kPa). The value of pressure adjustment is not a fixed value in each step. The value of pressure adjustment is fixed at 0.01 MPa, which is the uncertainty of the device measurement when traces of hydrate can be maintained for more than 2 h. The experimental uncertainty of measured *P_eq_* is ±0.005 MPa, the experimental uncertainty of the measured temperature is ±0.05 K.

## 6. Conclusions

This work explored the prediction of equilibrium hydrate formation conditions of HNGs-THF hydrate. A new thermodynamic model was proposed to predict the *P_eq_* of sII hydrate for CH_4_-helium-THF and applied in CH_4_-helium-CO_2_-THF hydrate. The effects of helium and THF on *P_eq_* were described in the proposed model. The model accurately can predict the *P_eq_* of CH_4_-helium-THF systems and CH_4_-helium-CO_2_-THF systems. The ARDs are less than 3% and GFs for HNGs-THF-water systems are more than 0.998.

The effect of helium on *P_eq_* is the dilution effect on CH_4_. Helium almost does not participate in the formation of hydrate. As the mole fraction of helium in the gas phase increases, helium dilutes the mole fraction of CH_4_ in the gas phase and reduces the fugacity of CH_4_, thus increasing the *P_eq_* of hydrate formation. A small Langmuir adsorption constant is used to represent the weak effect on *P_eq_* in the proposed model. The Langmuir adsorption of helium is 1.5% that of CH_4_ at 290.15 K

The effect of THF on *P_eq_* varies with the initial concentration of THF in the aqueous solution. There is no significant difference in the promotion effects of THF on *P_eq_* at high concentrations (15 wt%~19 wt%).

The new thermodynamic model and experimental data in this work can help to further understand the effects of helium and THF on *P_eq_* and can guide the prediction and control of HBGS of helium from HNGs. The other gas components of HNGs (like other hydrocarbons and nitrogen) play an important role in the hydrate formation process, which will be considered in our next work. This work showed the feasibility of HBGS, and the separation effect needs to be further experimented and studied in our next work.

## Figures and Tables

**Table 1:** The parameters of $C_{i}$ used for the model.

$C_{i}$	$X^{\prime }$ (Pa)	$Y^{\prime }$ (K)	$Z^{\prime }$ (K)
$C_{1}$ ($CH_{4}$)	6.2728 × 10^−15^	4879.29	23.01
$C_{2}(CO_{2})$	1.6464 × 10^−11^	2799.66	15.90
$C_{3}(helium)$	6.0000 × 10^−12^	2034.89	6.31

**Table 2:** The ARDs, GFs, and SDs for all the systems in this work.

Gases	Liquids	ARD	GF	SD
HNG1	5 wt% THF	1.7%	0.999	0.068
HNG2		2.5%	0.998	0.101
HNG3		2.7%	0.999	0.151
HNG1	10 wt%THF	1.8%	0.999	0.036
HNG2		2.6%	0.998	0.050
HNG3		2.7%	0.999	0.108
HNG1	19 wt% THF	1.7%	0.999	0.037
HNG2		2.6%	0.999	0.053
HNG3		2.5%	0.999	0.086
HNG4	5 wt% THF	2.3%	0.999	0.065
	10 wt% THF	2.8%	0.998	0.055
	19 wt% THF	2.9%	0.999	0.055

**Figure A1:**
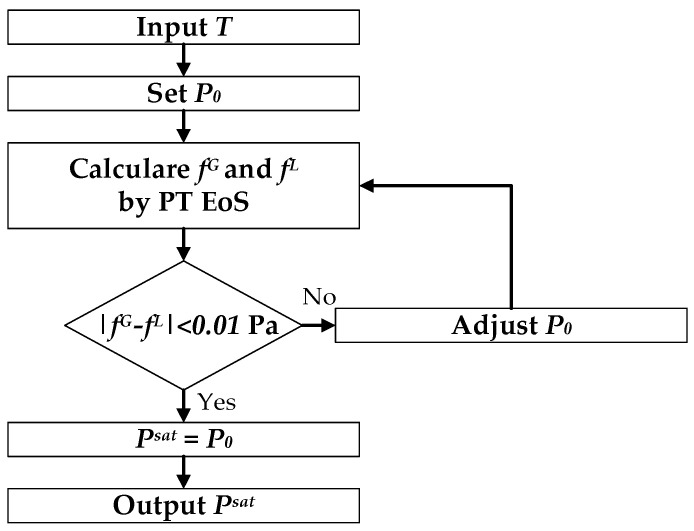
The pressure–temperature flash procedure for calculating $P_{2}^{sat}$. *f^G^* and *f^L^* are the fugacity of THF in gas and liquid phase in a pure THF system.

## Data Availability

Data are contained within the article.

## Nomenclature

Abbreviation	
ARD	average relative deviation
EoS	equation of state
GF	goodness of fit
HBGS	hydrate-based gas separation
HNG	helium-rich natural gas
NG	natural gas
PT	Patel–Teja
sI	structure I
sII	structure II
THF	tetrahydrofuran
Symbols	
$A^{\prime }$	the Antoine parameters of $f_{THF}^{H}$
$A_{i}$	the binary interaction parameter between gas *i* and THF in the hydrate
*a*	the parameters of PT EoS
$B^{\prime }$	the $\mathrm{Antoine}\;\mathrm{parameters}\;\mathrm{of}\;f_{THF}^{H}$
*b*	the parameters of PT EoS
$C^{\prime }$	the $\mathrm{Antoine}\;\mathrm{parameters}\;\mathrm{of}\;f_{THF}^{H}$
$C_{i}$	the Langmuir constant of gas *i*
*c*	the parameters of PT EoS
*F*	the constant associated with the gas molecule in the PT EoS
$f_{cor}$	*correction factor for* $f_{THF}$
$f_{i}$	the fugacity of CH_4_, CO_2_, and helium
$f_{THF}$	the fugacity of THF in the liquid phase
$f_{THF}^{H}$	the fugacity of THF in the basic hydrate
$P_{HNG}$	the *P_eq_* for HNG
$P_{{CH}_{4}}$	the *P_eq_* for pure CH_4_
*P_eq_*	thermodynamic equilibrium hydrate formation pressure
∆P%	the difference in *P_eq_*
$P_{2}^{sat}$	saturated vapor pressure of THF
*R*	the gas constant (8.314 J K^−1^ mol^−1^)
$v_{1}^{L}$	the mole volumes of water
$v_{2}^{L}$	the mole volumes of THF
$x_{1}$	the mole fractions of water
$x_{2}$	the mole fractions of THF
$X^{\prime }$	the $\mathrm{Antoine}\;\mathrm{parameters}\;\mathrm{of}\;C_{i}$
$y_{i}$	the mole fractions of gases in the gas phase
$Y^{\prime }$	the $\mathrm{Antoine}\;\mathrm{parameters}\;\mathrm{of}\;C_{i}$
$Z^{\prime }$	the $\mathrm{Antoine}\;\mathrm{parameters}\;\mathrm{of}\;C_{i}$
$\alpha_{THF}$	the activity of THF
$\alpha_{w}$	the activity of water
*β*	the parameter of hydrate structure
$\gamma_{1}$	the activity coefficients of water
$\gamma_{2}$	the activity coefficients of THF
*ζ*	the constant associated with the gas molecule in the PT EoS
$\theta_{i}$	the occupation fraction of the linked cages in hydrates filled by gas *i*
$\Lambda_{12}$	the parameter of Wilson model
$\Lambda_{21}$	the parameter of Wilson model
$\lambda_{12}-\lambda_{11}$	the parameter of Wilson model
$\lambda_{21}-\lambda_{22}$	the parameter of Wilson model
$\lambda_{1}$	the ratio of the linked-cage number to the water-molecule number
${\;\lambda }_{2}$	the ratio of the basic-cage number to the water-molecule number
*Δµ*	the difference in chemical potential
w	the mass fraction of THF

## Appendix A. Calculation for the Fugacity of Gases

The PT EoS to calculate the fugacity of CH_4_, CO_2_, and helium. It is expressed as follows [31]:

(A1) $$P=\frac{RT}{v-b}-\frac{a[T]}{v(v+b)+c(v-b)}$$

(A2) $$a(T)=\frac{\Omega_{a}\alpha (T)R^{2}T_{c}^{2}}{P_{c}}$$

(A3) $$\;b=\frac{\Omega_{b}RT_{c}}{P_{c}}$$

(A4) $$c=\frac{\Omega_{c}RT_{c}}{P_{c}}$$

(A5) $$\Omega_{a}=3{-3\zeta }^{2}+3\left(1-2\zeta \right)\Omega_{b}+\Omega_{b}^{2}+1-3\zeta$$

(A6) $$\Omega_{b}^{3}+\left(2-3\zeta \right)\Omega_{b}^{2}+3{-3\zeta }^{2}\Omega_{b}-\zeta^{3}=0$$

(A7) $$\Omega_{a}=1-3\zeta$$

(A8) $$\alpha ={\left[1+F\left(1-T_{\Gamma }^{0.5}\right)\right]}^{2}$$

where *ζ* and *F* are the constants associated with the gas molecule in the PT EoS, and the values are listed in Table A1.

**Table A1:** Parameters of PT EOS.

Gases	*ζ*	*F*
CH_4_	0.324	0.455336
CO_2_	0.309	0.707727
helium	0.329	0.452413
THF	0.313	0.733317

The $P_{2}^{sat}$ is calculated by pressure–temperature flash using PT EoS. The procedure is shown in Figure A1.
